# The Effect of Early Pediatric Rehabilitation in an Infant With Vitamin B12 Deficiency Associated With Developmental Delay: A Case Report

**DOI:** 10.7759/cureus.62648

**Published:** 2024-06-18

**Authors:** Pratiksha A Warghat, H. V. Sharath, Raghumahanti Raghuveer

**Affiliations:** 1 Department of Pediatric Physiotherapy, Ravi Nair Physiotherapy College, Datta Meghe Institute of Higher Education and Research, Wardha, IND; 2 Department of Neuro-Physiotherapy, Ravi Nair Physiotherapy College, Datta Meghe Institute of Higher Education and Research, Wardha, IND

**Keywords:** neurological development, b12 deficiency, infants, early physiotherapeutic intervention, developmental delays

## Abstract

Developmental delay is a multifaceted condition that can hamper a child's ability to attain developmental benchmarks within expected timelines. Vitamin B12 deficiency has been identified as a potentially reversible causative factor and is critical to neurological function, influencing myelination and nerve conduction. Insufficiency during critical developmental stages can lead to motor, cognitive, and language delays. Physiotherapy interventions have been found effective in addressing motor delays associated with both developmental delay and B12 deficiency. Early intervention programs that focus on motor skill development, sensory integration, and adaptive equipment use are among the interventions that physiotherapists provide. Collaboration with multidisciplinary teams allows physiotherapists to manage B12 deficiency effectively and provide rehabilitative strategies aimed at maximizing motor function and overall development for long-term health. Early identification and intervention in children with developmental delays is crucial, especially in cases related to vitamin B12 deficiency. Physiotherapy is a critical aspect of addressing motor delays associated with developmental delay and B12 deficiency. By providing early interventions, physiotherapists can help children attain their full potential and attain developmental milestones. In conclusion, this highlights the significance of early identification and intervention in children with developmental delay, especially those with vitamin B12 deficiency, for optimal long-term health.

## Introduction

Vitamin B12 is an essential nutrient that performs various functions in the human body, including hematopoiesis, metabolism, growth, and early brain development. It is only found in animal-derived foods such as meat, fish, eggs, and dairy products, meaning that individuals who follow a vegetarian diet are at higher risk of B12 deficiency [1]. Typical symptoms of B12 deficiency include megaloblastic anemia and sensory-motor abnormalities. Severe B12 deficiency can cause neurological symptoms in infants, including developmental regression, apathy, and skin pigmentation changes [2]. B12 deficiency may result from insufficient intake, malabsorption, or underlying health issues. In children, B12 deficiency can have profound effects on neurological development. Since vitamin B12 is crucial for the formation and maintenance of myelin, the protective sheath surrounding nerves, its deficiency can lead to disruptions in nerve signaling and overall neurological function [3].

For infants affected by B12 deficiency, the initial symptoms include irritability, feeding difficulties, and aversion to foods other than breast milk. They may also experience spitting and fail to thrive. Pallor and early darkening of the skin, particularly around the knuckles, may become noticeable. As the condition progresses, it affects neurodevelopment, causing infants to lag behind their peers and experience anemia and skin pigmentation changes. If left untreated, multiple symptoms of developmental regression may occur, sometimes triggered by intercurrent illness [4-6]. The infants may appear lethargic but become irritable when disturbed. Apathy, a vacant stare, and an open mouth with drooling are characteristic of this stage of neuro-regression. Microcephaly and hypotonia with brisk reflexes are commonly noted [7-9].

A prolonged deficiency of vitamin B12 can affect the production of myelin, a protective covering that surrounds nerve cells and helps in communication. Vitamin B12 is present in two forms - adenosylcobalamin and methylcobalamin - which act as cofactors in enzymatic reactions that impact myelin formation. A deficiency in either of these forms can lead to impaired myelination or even cause demyelination. Although severe nutritional vitamin B12 deficiency can lead to abnormal neural myelination or demyelination, the rapid improvement upon initiation of vitamin B12 therapy does not result from morphological change [10,11]. Symptoms like apathy, muscle hypotonia, anorexia, and involuntary movements of the limbs and tongue may resolve within days of therapy.

Vitamin B12 deficiency can affect neurodevelopment by altering the S-adenosylmethionine:S-adenosylhomocysteine (SAM:SAH) ratio, which affects methylation reactions essential for synthesizing proteins, lipids, and neurotransmitters in the brain. Elevated homocysteine levels, which occur due to deficiency, can cause neurodegenerative diseases. The decrease in the SAM:SAH ratio also hinders DNA synthesis and cell division [12-14].

The connection between developmental delay and B12 deficiency lies in the impact of B12 on neurological development. When a child's body lacks an adequate supply of vitamin B12, the nervous system may not develop properly, leading to a range of neurological symptoms that can contribute to developmental delay. Physiotherapy plays an important role in developmental delay associated with B12 deficiency. A tailored physiotherapeutic intervention approach is applied while treating this condition which includes the combination of neurodevelopmental therapy, sensory integration therapy, fine motor activity training, and play therapy. These therapeutic exercises are designed to target specific motor skills, improve muscle strength and coordination, and enhance overall physical function. These may include activities to improve balance, gait training, and exercises to promote fine motor control. Sensory integration techniques may be utilized to address sensory processing difficulties, helping children better understand and respond to sensory input from their environment.

## Case presentation

We present a case of a one-year-old male infant who was brought to the pediatric outpatient department at Acharya Vinoba Bhave Rural Hospital with his caregiver (mother). The caregiver complained about a high-grade fever, cold, and cough, along with a delay in achieving milestones for the past six months and poor activity. The patient was admitted to the pediatric ward and underwent medical investigations including an MRI and blood tests. The MRI revealed atrophy in the fronto-parieto-temporal region coupled with mild thinning of the corpus callosum in the body and splenium. Upon blood investigation, the infant was diagnosed with a vitamin B12 deficiency. The doctor prescribed medication and B12 injections and referred the infant to physiotherapy due to delayed milestone achievement.

Clinical presentation

Before the assessment, consent was obtained from the primary caregiver (mother). On observation, the patient was found to be sitting and conscious, hemodynamically stable with a distinct ectomorphic build, and low overall activity. During respiratory examination, air entry was bilaterally equal. The assessment of developmental milestones revealed that the child was unable to sit, crawl, and creep. Fine motor and reaching for objects were achieved at one year of age. In the evaluation of developmental reflexes, it was found that the spinal, brain stem, and cortical were integrated and tilt reactions were absent. On motor examination, a passively complete range of motion was noted. According to the tone grading scale, the upper limb tone was 2+. Furthermore, deep tendon reflexes for the upper and lower limbs were 2+ (normal response). No tightness was present in the bilateral upper and lower limb. The anthropometric measures are described in Table 1.

**Table 1:** Anthropometric measures Anthropometric measurements were noted on the date of assessment and were compared to the normal values.

Anthropometric measures	At present	Normal
Length	69 cm	78.5 cm
Weight	8.7 kg	10.5 kg
Head circumference	43 cm	46 cm
Chest circumference	49 cm	48-50 cm

Developmental milestone

At one month, the child could hold and maintain head up while on his stomach or when held upright; by five months, he could roll from stomach to back and vice versa; and by 11 months, he could sit steadily without support. However, he had not yet achieved milestones such as crawling on hands and knees or pulling up to stand, as mentioned in Table 2.

**Table 2:** Developmental milestones

Gross motor	Procedure	Normal attaining months	Attained months
Head control	While on the stomach or when held at the shoulder maintains a constant head position in the middle of the body	6 weeks	1 month
Rolling	Rolls from stomach to back and back to stomach	4-6 months	5 months
Sitting	Sits steadily without support	5-7 months	11 months
Creeping	Creeps on the stomach	6-8 months	Not achieved
Crawling	Crawling on hands and knees	9-11 months	Not achieved
Standing	Self-pulls to stand while clinging to furniture	9-12 months	Not achieved
Walking	Holds onto the furniture while moving	10-15 months	Achieved

Fine motor milestones

At four months, he could grasp objects; at five months, he could reach for objects; at six months, he could drop objects and bring toys to his mouth; at 12 months, he could transfer objects between hands but had not yet achieved advanced grasps or spontaneous scribbling, as mentioned in Table 3.

**Table 3:** Fine motor milestones

Fine motor	Procedure	Normal attaining months	Attained months
Grasp reflex	Grasps object placed in hands	0-3 months	4 months
Reach	Reaches for objects placed in the midline with both hands	2-4 months	5 months
Release	Drops objects held in hands	3-6 months	6 months
Mouthing	Takes toys and objects to the mouth	3-6 months	6 months
Transfers	Transfers objects from one hand to another	4-6 months	12 months
Grasp	Holds objects using the thumb side of the hand	6-8 months	Not achieved
	Holds objects like marbles between thumbs first two fingers	8-10 months	Not achieved
Scribbling	Scribbles spontaneously	10-11 months	Not achieved

Primitive reflex

Reflexes emerged and integrated progressively. Supine and prone reflexes were integrated, four-point kneeling reflex was integrated, but the sitting reflex and standing reflex were present, as mentioned in Table 4.

**Table 4:** Reflexes - tilt reaction

Reflex	Emerges at	Integrated/present
Supine and prone	6 months	Integrated
Four-point kneeling	7-12 months	Integrated
Sitting	9-12 months	Present
Standing	12-18 months	Present

Investigation 

**Figure 1:**
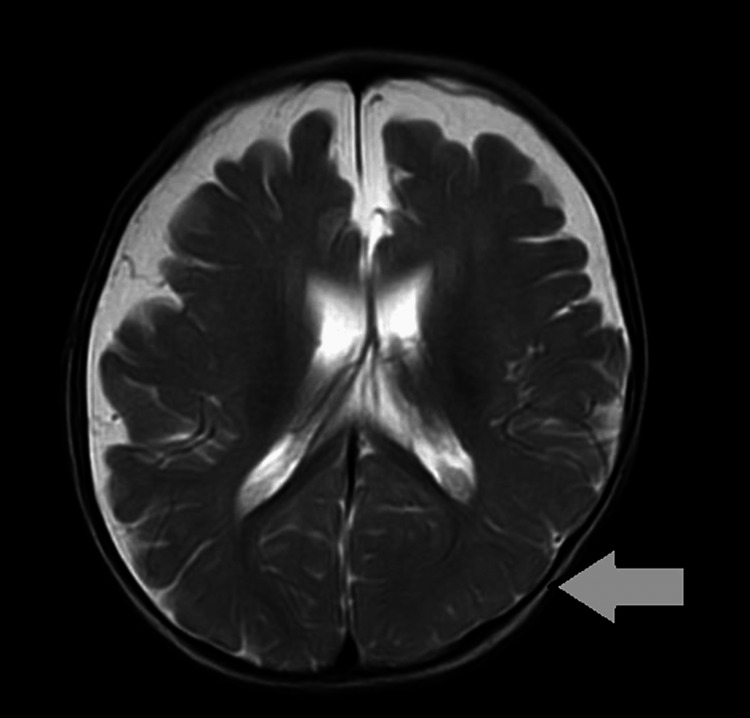
MRI scan The scan showed atrophy in the fronto-parieto-temporal region, coupled with mild thinning of the corpus callosum in the body and splenium.

The MRI results indicated atrophy in the fronto-parieto-temporal region, which refers to a decrease in size or loss of brain tissue in these specific areas. This can be associated with various neurological conditions such as Alzheimer's disease, frontotemporal dementia, or other neurodegenerative disorders. Additionally, there was mild thinning of the corpus callosum in the body and splenium. The corpus callosum is a bundle of nerve fibers that connects the two hemispheres of the brain, and thinning of this structure can sometimes indicate neurological issues as well. These findings may require further evaluation by a neurologist or other healthcare professional to determine the underlying cause and appropriate management. The MRI scan is shown in Figure 1.

Outcome measures

Table 5 compares the scores before and after rehabilitation for outcome measures, including GMFM (Gross Motor Function Measure), INFANIB (Infant Neurological International Battery), and Hammersmith Infant Neurological Examination. It shows significant improvements across all measures after rehabilitation.

**Table 5:** Outcome measures GMFM: Gross Motor Function Measure; INFANIB: Infant Neurological International Battery

Outcome measures	Pre rehabilitation score	Post rehabilitation score	Normal scoring
GMFM	24.7%	63.2%	100%
INFANIB	62	81	>83
Hammersmith Infant Neurological Examination	50	71	67-68

Physiotherapeutic intervention

**Figure 2:**
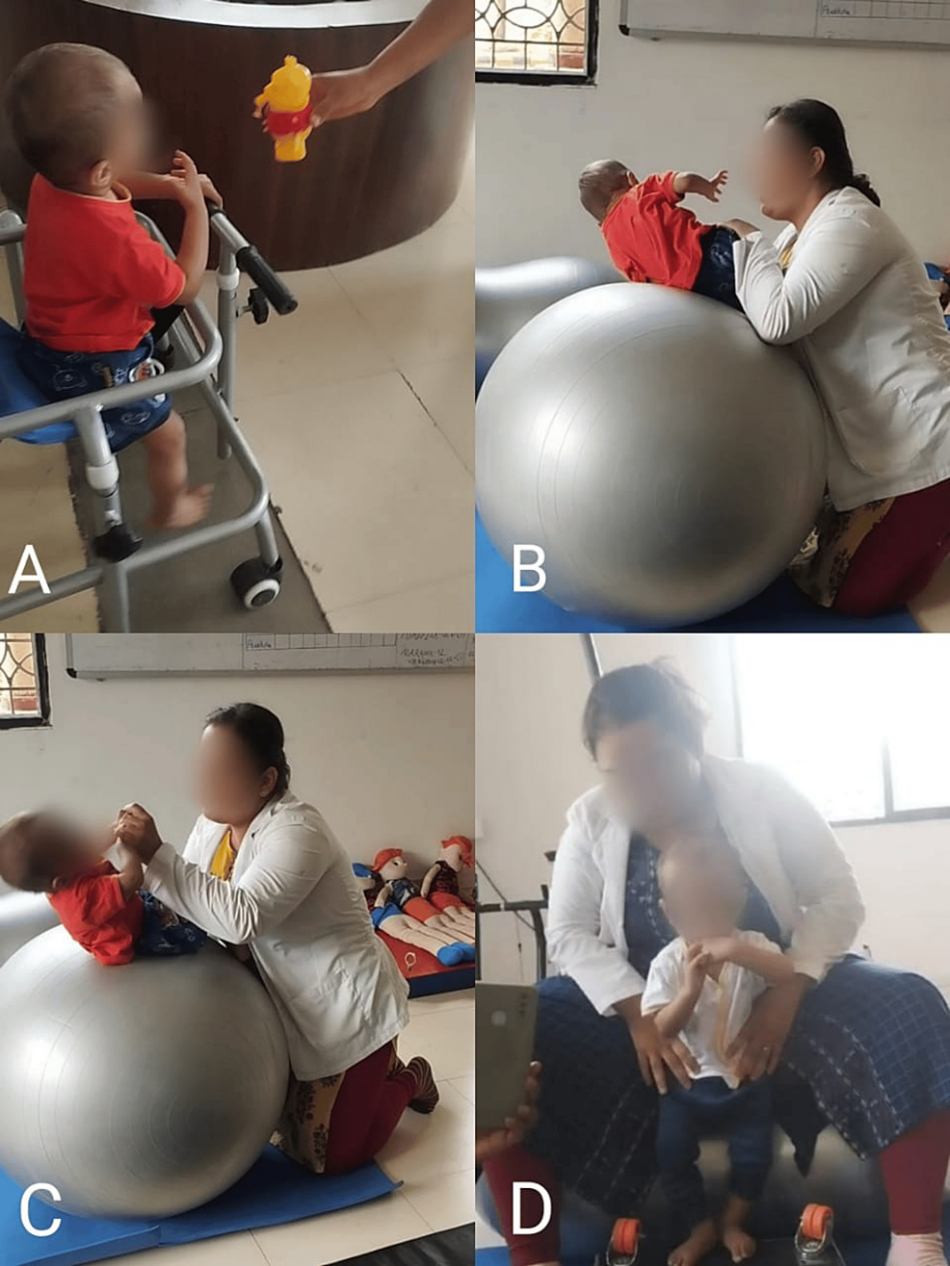
Physiotherapeutic intervention (A) Walking with the assistance of a walker with sensory stimulation; (B) Trunk control on Swiss ball without assistance; (C) Trunk control with assistance; (D) Initiating sit to stand motion with assistance

Table 6 and Figure 2 depict physiotherapeutic intervention, including caregiver education, neurodevelopmental approach, and sensory integration techniques.

**Table 6:** Physiotherapeutic intervention

Problem list	Short terms goal	Intervention
Caregiver education	Educating caregiver	Educate caregivers on B12 deficiency leading to developmental delay management, medication, and therapeutic exercise. Teach proper positioning and handling techniques and environmental modifications, and promote engagement and interaction. Caregivers receive emotional support and are empowered to advocate for their child's needs. Physiotherapists maintain ongoing communication and collaboration with caregivers and other healthcare professionals to optimize the child's development and well-being.
Difficult to control trunk	To develop trunk control	Neurodevelopmental treatment (NDT) approach for trunk control on a Swiss ball: the child lies in a supine position on a Swiss ball, holds the therapist's hand, and comes to a sitting position. Trunk control on swinger: in sitting and standing positions with forward and backward motion.
Difficulty in standing	To initiate standing	NDT approach on a Swiss ball: the infant is made to stand on a Swiss ball while the therapist is in a sitting position on the Swiss ball and supports the infant's knee and trunk from the back side, which initiates weight bearing and weight shifting on the feet to provide vestibular stimulation and maintain standing balance with support. NDT approach on swinger: the therapist supports the child and stabilizes the knee, and weight bearing is initiated.
Difficulty in walking	To initiate walking with support	The therapist from behind the baby holds the trunk and with auditory and visual stimulation tries to initiate walking. Walker assisted walking with auditory and visual stimulation.

## Discussion

Various factors like pre-term delivery, post-term delivery, meconium aspiration, low birth weight, neonatal complications in the child, no pre-lacteal feeds given, complications in the mother during delivery, and birth injury cause developmental delays in children, and among them, vitamin B12 deficiency plays a significant role [15]. B12 is essential for neurological development, and its deficiency can lead to delays. This deficiency can result from different causes such as inadequate dietary intake, malabsorption issues like pernicious anemia or gastrointestinal disorders, and genetic factors. When B12 levels are insufficient, it affects the nervous system's development, resulting in cognitive, motor, and language delays.

Children with B12 deficiency and developmental delay struggle with memory, attention, learning, and exhibit delays in motor skills like sitting, crawling, and walking, and may experience delayed speech and language acquisition. Moreover, behavioral issues such as irritability and lethargy might surface. Diagnosis involves blood tests; treatment typically consists of B12 supplementation through injections or oral supplements. Prevention focuses on ensuring children receive adequate B12 through their diet or supplementation, especially for those at risk due to breastfeeding from deficient mothers or medical conditions affecting absorption. Vigilance from parents and healthcare providers is essential to recognize and address developmental delays promptly, thus supporting healthy neurological development. Physiotherapy plays an important role in treating developmental delays associated with B12 deficiency [16-19].

A tailored physiotherapeutic intervention approach is applied while treating this condition, which includes a combination of neurodevelopmental therapy, sensory integration therapy, fine motor activity training, and play therapy [20-22]. These therapeutic exercises are designed to target specific motor skills, improve muscle strength and coordination, and enhance overall physical function. They may include activities to improve balance, gait training, and exercises to promote fine motor control. Sensory integration techniques may be utilized to address sensory processing difficulties, helping children better understand and respond to sensory input from their environment. This can include activities aimed at improving proprioception and tactile stimulation. From a physiotherapy standpoint, developmental delay stemming from B12 deficiency poses unique challenges that require specialized intervention.

B12 deficiency can significantly impact a child's motor and neurological development, affecting their ability to reach developmental milestones. Physiotherapists play a critical role in addressing these delays through tailored interventions. It aims to improve motor development by targeting gross motor skills such as sitting, crawling, standing, and walking. Therapists employ exercises and activities that promote strength, coordination, and balance, helping children achieve these milestones.

## Conclusions

Early physiotherapy intervention plays a crucial role in addressing developmental delay caused by B12 deficiency. This condition presents significant challenges and requires specialized attention from the healthcare team and primary caregiver. Tailored interventions targeting motor and neurological development, including the neurodevelopmental treatment (NDT) approach, sensory integration, and play therapy, can help children with B12 deficiency overcome delays and reach their developmental milestones. Effective treatment involves early intervention, collaboration with other healthcare professionals, and family education and support. By addressing all these aspects comprehensively, physiotherapists can empower children and their families to navigate the challenges of B12 deficiency and achieve optimal long-term health and well-being.
